# Electrocardiography in Early Diagnosis of Cardiovascular Complications of COVID-19; a Systematic Literature Review

**DOI:** 10.22037/aaem.v9i1.957

**Published:** 2020-12-17

**Authors:** Reza Nemati, Mahasty Ganjoo, Faezeh Jadidi, Ahmad Tanha, Reza Baghbani

**Affiliations:** 1 Department of Medical Emergencies, School of Allied Medical Sciences, Bushehr University of Medical Sciences, Bushehr, Iran.; 2 Student Research Committee, Zarand School of Nursing, Kerman University of Medical Sciences, Kerman, Iran.; 3 Clinical Research Development Center, The Persian Gulf Martyrs Hospital, Bushehr University of Medical Sciences, Bushehr, Iran.

**Keywords:** COVID-19, Heart Diseases, Heart Injuries, Electrocardiography, Prognosis

## Abstract

**Introduction:**

Many reports have stated that patients admitted for COVID-19 may also suffer from cardiovascular diseases, suggesting cardiovascular involvement in COVID-19. Since there is direct association between electrocardiography (ECG) data and the prognosis of cardiovascular disease, a systematic literature review was performed in the present study to address this association and make a conclusive agreement on the early diagnostic and prognostic values of ECG in patients with COVID-19.

**Methods:**

Electronic databases including PubMed, Scopus, Web of Science, Science Direct, Ovid, Embase, and Google Scholar were searched for “COVID-19” and “ECG” using all their equivalents and similar terms as search words. Afterwards, the records were limited to English articles and irrelevant documents, as well as articles that reported drug-induced cardiac dysfunction or patients with previous history of cardiovascular complications were excluded.

**Results:**

Overall, 31 articles with 2379 patients were found and used for qualitative data extraction. Findings showed that there is a significant association between COVID-19 infection and ECG findings. Also, ST-segment changes, T wave inversions, QT prolongation, and atrial fibrillation were found to be early indicators of cardiac involvement of COVID-19, which were associated with worse outcomes.

**Conclusion:**

It is recommended to use ECG as a valuable diagnostic and prognostic tool for cardiac evaluation of patients with COVID-19.

## Introduction

Coronavirus Disease 2019 (COVID-19) pandemic started from Wuhan, China in 2019 (1). Coronavirus can cause serious damage to the liver, kidneys, nervous system, lungs, etc. in humans (2-5). Although the lungs are the main target of the virus causing COVID-1, the virus may also affect the heart (6). Although the effects of coronavirus infection on the circulatory system have not been fully understood, it is thought that the new coronavirus may exacerbate the problems of patients with heart failure (7). In addition, there are many reports of high rates of acute heart injury, arrhythmia (irregular heartbeat), hypotension, and tachycardia and other cardiovascular diseases among patients admitted to the intensive care unit due to COVID-19 (8). Overall, by 15 November 2020, 53.7 million confirmed cases and 1.3 million deaths have been reported to WHO (9). Coronavirus aggravates cardiac complications in patients with cardiovascular disease, such that mortality rates of about 10.5% have been observed in patients with cardiovascular diseases (10).

According to recent reports, most patients admitted for COVID-19 have cardiovascular or cerebrovascular diseases. Findings have shown that myocardial injury is associated with adverse prognosis. On the other hand, recent evidence has suggested cardiovascular involvement in COVID-19. There is also a correlation between electrocardiography (ECG) data and the severity of cardiovascular diseases, and clinical outcomes such as death. Therefore, it is suggested that ECG can be considered as an important diagnostic tool in the early diagnosis of cardiovascular complications, particularly in patients with COVID-19 infection (11). Moreover, ECG changes, as the typical sign of cardiac injury, may reflect a cardiac abnormality in patients with COVID-19, and may also add important prognostic information (12). Accordingly, it is thought that the risk of mortality and cardiovascular comorbidities can be predicted in patients with COVID-19 by evaluating ECG data and markers of myocardial injury (13). 

This study may not completely include or reflect the ECG findings of all the reported cases with COVID-19; however, the main aim of this study was to emphasize the cardiac complications due to novel coronavirus disease and the necessity and importance of ECG findings as a prognostic marker. The aim of this study was to determine whether performing an ECG on presentation to hospital has diagnostic value or can provide additional prognostic information, specifically regarding severity of the disease or death outcome, or not. The results of this review may provide a conclusive statement on the value of ECG in patients with COVID-19 infection.

## Methods

***Study search and inclusion criteria***

In the present study, a systematic literature review was performed in Medline via PubMed, Scopus, Web of Science, Science Direct, Ovid, Embase, and Google Scholar to investigate the predictive role or diagnostic value of ECG in patients with COVID-19 infection. For this purpose, “Electrocardiography” and “COVID-19” with all their equivalents and different written forms as key terms were searched in the PubMed as follows: (Electrocardiogram OR ECG OR electrocardiography) AND (Coronavirus OR Coronavirus Disease 2019 OR COVID-19 OR COVID 19 OR SARS-CoV-2 OR COVID OR Corona). To minimize the risk of bias, no strict inclusion criteria were applied and almost all relevant documents were included for data synthesis. First, the search was limited to English articles, and then, review articles, book chapters, letters, and conference papers were excluded. In the Scopus, ECG with all its similar terms was searched in the title, keyword, and abstract. Then, Covid-19 was searched within the results. Afterwards, irrelevant articles were excluded from further evaluation. Chloroquine-induced cardiac complications or other drug-induced ECG variations were excluded and only ECG changes due to coronavirus infection were enrolled for further assessment. Also, reports of patients with documented underlying cardiovascular (CV) comorbidities, coronary artery disease (CAD), or cardiac complications were excluded from additional assessment. The search was independently performed by authors, and any disagreement between the authors in each step was resolved by other authors through repeating the search. PRISMA checklist 2009, which is a valid protocol for performing systematic reviews, was used for study design and in the article selection process (14). According to this protocol, the study was designed to answer the question “can ECG predict the outcomes of patients with COVID-19 and what are the early and frequent alterations found in ECG?”. To follow this protocol, all ECG findings were noted and described.

***Risk of bias assessment***

Systematic reviews only rely on the data reported by other studies; hence, the risk of bias in the included studies should be assessed in reporting systematic reviews. Accordingly, in the present systematic review, the risk of bias was evaluated based on the guideline of Cochrane assessing method for observational studies (15). According to this method, (−) showed high risk of bias, whereas (+) showed low, and (?) indicated unclear risk of bias.

***Data synthesis and the variables***

All necessary information for data extraction, including the first author’s name, study type, publication date, and number of patients, as well as their sex and mean age were extracted and summarized in Table 1. Furthermore, as the main outcome, ECG results were collected and used for qualitative data description. 

## Results

**Figure 1:**
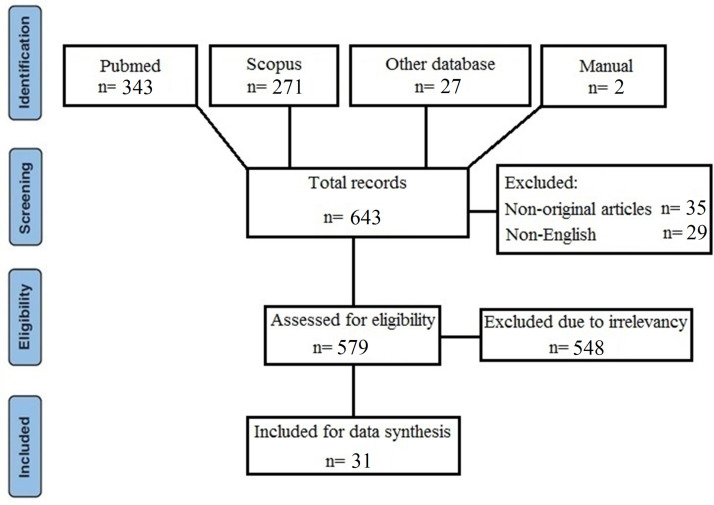
Article selection process

Literature search was performed on August 2020. Of the 641 articles found in database search, 343 were in the PubMed, 271 were in Scopus, and 27 were in other databases. Also, 2 additional articles were found through reference list search of the included articles. After exclusion of irrelevant articles, a total of 31 related articles were found, 2 of which were retrospective, 3 were cross-sectional, 2 were case control, one was a prospective cohort, and 23 were case reports. The process of article selection for final evaluation is demonstrated in Figure 1. Overall, 2379 patients were enrolled via the 31 included articles, wherein 1575 were male, and 804 were female. All of the articles were published in 2020. The age group of patients varied between 8 to 92 years. The length of follow up in all studies was during hospitalization (2 weeks). The result of risk of bias assessment for the included studies is presented in Figure 3. Demographic information and the most important findings of the included articles are shown in Table 1.

**Figure 2:**
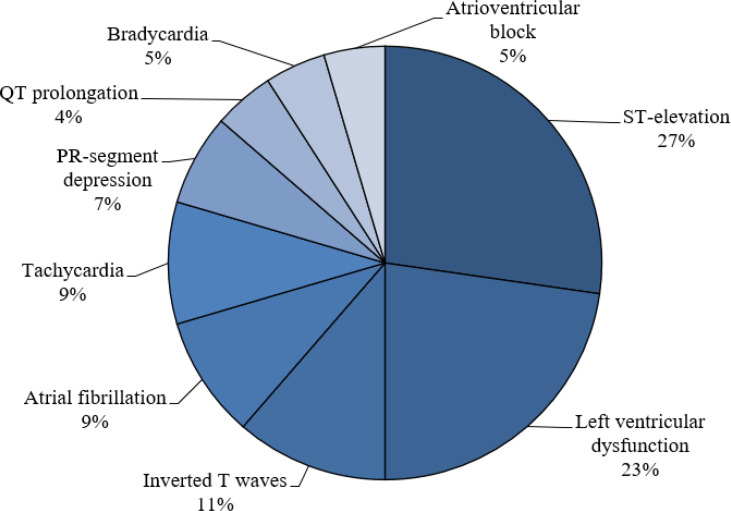
Frequency of electrocardiography complications among patients with COVID-19 infection

According to reports, many patients with COVID-19 had abnormal ECG, including atrial fibrillation, ST-T segment changes, tachycardia, bradycardia, hypertension, atrial premature contractions, intraventricular block, and right bundle branch block (16, 17). It was also demonstrated that mean QTc (410.8 ± 24.3 msec vs. 394.6 ± 20.3 msec, p < 0.001), median QTd (47.52 vs. 46.5) and Tpe/QTc (0.19 ± 0.02 vs. 0.18 ± 0.04, p = 0.036) were significantly higher in patients hospitalized for COVID-19 compared to the control group (18). Findings of a cross-sectional cohort showed abnormal PR interval behavior and increase in heart rate, which was associated with increased risk of death and need for endotracheal intubation (19). Also, ECG revealed Brugada-like ECG pattern with normal coronary arteries in some patients with COVID-19 (20, 21). Patients with the new coronavirus disease who passed away had abnormal ECG including left- and right-sided heart disease at the time of infection and their cardiac dysfunction led to acute kidney injury with anuria, multiorgan failure, acidosis, and hemoptysis (17, 22). On the other hand, the results of a study demonstrated that only 12.5% of patients with Covid-19 showed cardiac complications, and concluded that typical signs of myocarditis were not observed on ECG in these patients (23). On the contrary, findings of a large prospective study revealed that over 55% of patients with COVID-19 infection had an abnormal ECG such as left and right ventricular complications with evidence of myocardial infarction (24). Frequency of each ECG complication is demonstrated in Figure 2. 

**Figure 3:**
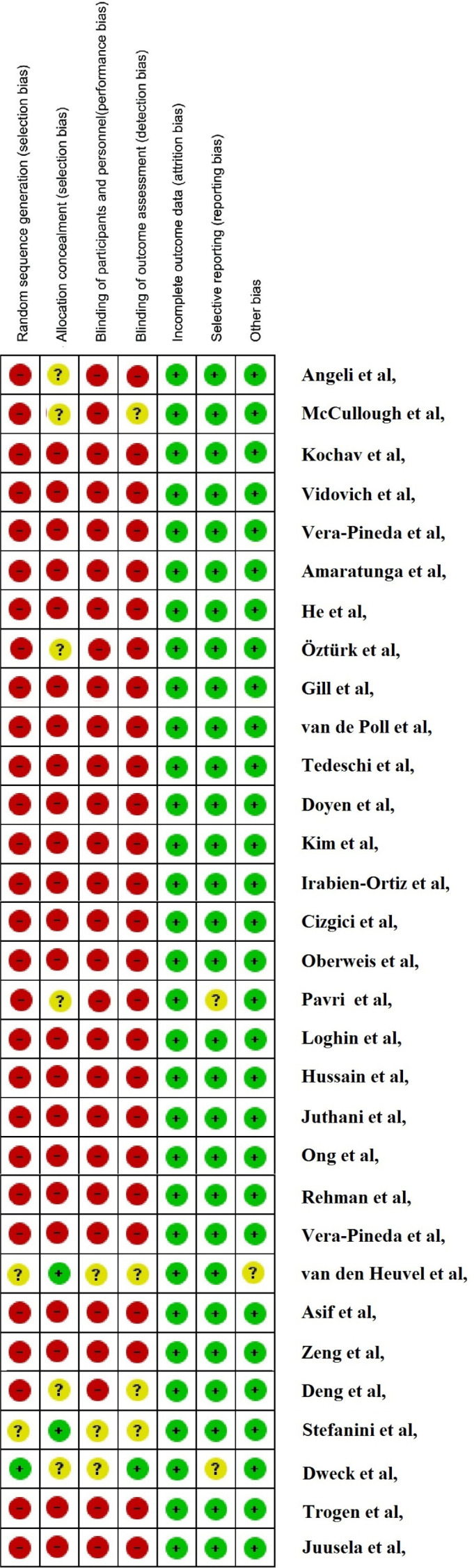
Risk of bias assessment for included articles based on Cochrane risk of bias evaluation method

**Table 1:** General information of included studies

No.	Author	Country	Study type	No. of Patients	Mean age	Male/Female ratio	ECG findings
1	Angeli F, (16)	Italy	CCS	50	64	36/14	Atrial fibrillation, ST-T changes, tachy-brady syndrome
2	McCullough SA, (17)	USA	RS	756	63.3	476/280	Atrial premature contractions, intraventricular block, repolarization, right bundle branch block
3	Kochav SM, (25)	USA	CR	4	56, 64, 70, 76	3/1	Atrioventricular dissociation, high-grade heart block, atrial fibrillation, T wave inversions and QT prolongation, sinus tachycardia with left bundle branch block
4	Vidovich MI, (21)	USA	CR	1	61	1/-	Brugada-like ECG pattern with normal coronary arteries
5	Vera-Pineda R, (22)	Mexico	CR	4	26, 64, 66, 76	4/-	Ventricular dysfunction
6	Amaratunga EA, (26)	USA	CR	4	55, 60, 73, 78	2/2	Bradycardia
7	He J, (12)	China	CR	2	66, 70	1/1	Atrioventricular block, ST-segment elevation, ventricular tachycardia
8	Öztürk F, (18)	Turkey	CCS	51	49.2	29/22	High QTc, QTd, and Tpe/QTc
9	Gill GS, (27)	USA	CR	2	34, 65	-/2	ST elevations, Low amplitude, PR segment depressions
10	van de Poll SWE, (20)	Netherlands	CR	2	40, 58	2/-	Sinus rhythm
11	Tedeschi D, (28)	Italy	CR	1	60	1/-	ST-elevation, myocardial infarction
12	Doyen D, (29)	France	CR	1	69	1/-	Left ventricular hypertrophy, diffuse inverted T waves
13	Kim IC, (30)	Korea	CR	1	21	-/1	Severe left ventricular systolic dysfunction
14	Irabien-Ortiz Á, (31)	Spain	CR	1	59	-/1	Concentric hypertrophy, Reduced intraventricular volumes, ST-segment elevation, PR-segment depression, low voltages
15	Cizgici AY, (32)	Turkey	CR	1	78	1/-	Atrial fibrillation and ST elevation
16	Oberweis ML, (33)	Belgium, Luxembourg	CR	1	8	1/-	LVEF
17	Pavri BB, (19)	USA	CSS	75	67	37/38	Paradoxical prolongation (abnormal PR interval) or lack of shortening
18	Loghin C, (34)	USA	CR	1	29	1/-	ST-segment elevations
19	Hussain H, (35)	USA	CR	1	51	1/-	Global left ventricular hypokinesia, reduced LVEF
20	Juthani P, (36)	USA	CR	1	29	1/-	ST-elevations
21	Ong E, (37)	USA	CR	1	29	1/-	ST-elevations
22	Rehman M, (38)	USA	CR	1	39	1/-	ST elevations, and T-wave inversion
23	Vera-Pineda R, (22)	Mexico	CR	4	26, 64, 66, 76	4/-	LVEF
24	van den Heuvel FMA, (39)	Netherlands	CSS	51	63	40/11	LVEF
25	Asif T, (40)	USA	CR	2	64, 71	1/1	ST elevation, non-specific T wave changes
26	Zeng JH, (41)	China	CR	1	63	1/-	LVEF
27	Deng Q, (23)	China	RS	112	65 (24-92)	57/55	Pericardial effusion
28	Stefanini GG, (42)	Italy	CSS	28	68	20/8	ST-elevation
29	Dweck MR, (24)	USA	PCS	1216	62	851/365	LVEF, RVEF
30	Trogen B, (43)	USA	CR	1	17	1/-	Tachycardia and T-wave inversion
31	Juusela A, (44)	USA	CR	2	26, 45	-/2	LVEF, hypokinesis

CCS: case control study; RS: retrospective study; CR: case report; CSS: cross-sectional study; PCS: prospective cohort study; ECG: electrocardiography; LVEF: Left ventricular ejection fraction; RVEF: Right ventricular ejection fraction.

## Discussion

Findings indicated that ST-elevation, bradycardia, atrial fibrillation, left ventricular ejection fraction, and T-wave inversion could be a possible predictor of worse outcome of COVID-19 (26). The results of many studies indicate that infectious diseases cause changes in the ECG, which can indicate cardiovascular damage in these patients (45-50). During the SARS pandemic, a study by Tak ‐ Sun et al. (2004) showed that pneumo-mediastinum complication in SARS can be a possible cause of chest pain and ECG changes (45). The results of yu et al. study (2006) also indicated that cardiovascular complications including hypotension and tachycardia were common in patients with SARS (46). Also, the results of a 24-hour ECG study of patients with diphtheria showed that many arrhythmias were recorded in all patients (47). In a meta-analysis on Chagas disease, Rojas et al. (2018) also concluded that many changes and arrhythmias such as right bundle branch block (RBBB), left anterior fascicular block (LAFB), atrioventricular (AV) block, atrial fibrillation (AF), and Flutter are observed in the disease. Also, the prevalence of ECG changes in children is the same as in adults (48). Also, studies on other diseases such as hemorrhagic fever with renal syndrome (49) and Lyme disease(50) show the high importance of ECG findings in the diagnosis and cardiac complications of these patients. Moreover, studies reported that several ECG indicators such as QTc, QTd, and Tpe/QTc increased in patients with COVID-19, indicating cardiomyocyte involvement in these patients (18). Clinical evidence also indicated that abnormal ECG in patients with COVID-19 may reflect a wide range of cardiovascular complications, and these patients may be at higher risk of death (16, 17). Recent data have shown that myocarditis and acute cardiac injury are common presentations, observed in almost one fifth of patients with COVID-19, with overall 50% survival rate, suggesting the necessity of performing ECG in cases of COVID-19 (29-31). Impaired ventricular function in young patients and children also indicates that the clinicians should be aware of the cardiac involvement in suspected cases of COVID-19 (33).

The results of studies also demonstrated that myocardial dysfunction may be a direct manifestation of COVID-19, since cardiac complications were also evident in patients with no previous history of cardiovascular disease (22). Recent data have shown that coronavirus can also affect the small or microvascular vessels and the large coronary arteries even in healthy individuals with no history of cardiovascular diseases. Findings have previously shown that hydroxychloroquine, chloroquine, and azithromycin were also associated with QTc prolongation, but epidemiological data revealed that unlike drug-induced arrhythmias, which are temporary, cardiovascular diseases due to coronavirus may lead to fatal cardiac dysfunction (51, 52). Also, impaired left- and right-ventricular functions were found to be significantly associated with high mortality among patients with COVID-19 (53). Consistent with these findings, the results of an animal study showed that coronavirus infection can lead to sudden cardiac death by increasing ventricular vulnerability in the absence of severe clinical signs of congestive heart failure (54). 

Although several studies have shown that vaccination against the ‎Influenza virus can reduce the risk of myocardial infarction, yet there is no information on the mechanistic association between coronavirus and cardiac dysfunctions (55). Animal studies on SARS-CoV have previously shown a connection between virus-induced pulmonary infections and myocardial disease (43). These data showed that SARS-CoV-2 probably has a direct effect on myocardial ion channels (56), while recent data suggested that the etiology of cardiac dysfunction due to SARS-CoV-2 seems to be multifactorial and cannot be through direct damage by the virus, and it is probably due to the response of the host’s immune system to the viral infection or excessive cytokine release (33). Consistent with these findings, clinical data have also suggested that myocardial injury is probably one of the systemic complications of the new coronavirus (23). Also, T-cell dysregulation, microvascular damage, hypotension and hypoxia-induced myocardial injury are considered as other most probable mechanisms for cardiac injury due to COVID-19 (57). Also, ECG changes in the severe stages of COVID-19 have been found to be the consequences of virus-induced hypoxia and inflammatory damage (12, 26).

The major limitation of this review is lack of enough population-based evidences on the diagnostic value of ECG in complementary and cost-effective diagnosis of COVID-19 infection, since COVID-19 pandemic crisis has affected the priority of the global health system. On the other hand, these results may not be sufficient to reach a conclusive statement about the diagnostic and prognostic values of ECG, because it is not long since this pandemic has emerged; however, findings of this study may encourage clinicians and health practitioners to consider ECG in patients with COVID-19. Clinical data regarding ECG characteristics of patients with COVID-19 is limited. Therefore, further research and clinical observation of these patients is critical to clarify the importance and prognostic value of ECG characteristics in predicting morbidity and mortality.

COVID-19 has been proven to be the leading cause of acute coronary syndrome, myocarditis, and heart failure in both the pediatric and adults (35, 40, 41, 43). Also, it has been demonstrated that cardiomyopathy is a frequent finding in pregnant women with COVID-19 (44, 58). According to the ECG results of a large prospective study, more than half of all suspected cases of COVID-19 showed cardiac complications (24). A growing body of clinical data has shown that COVID-19 may be associated with a high rate of cardiac diseases, leading to a high rate of mortality. Altogether, clinical evidence suggested that ST-segment elevation and T wave inversions, in the absence of other cardiovascular risk factors such as cardiac troponin, can be interpreted as early signs of COVID-19–related myocarditis, suggesting the necessity of early and rapid ECG testing in suspected cases of COVID-19 to prevent severe cardiovascular complications and death (34).

## Conclusion:

In sum, the results of studies demonstrated that COVID-19 can directly or indirectly lead to heart complications even in the absence of other cardiovascular risk factors. Also, it was found that dynamic ECG changes, particularly left and right ventricular dysfunction, ST-segment elevation, and T wave inversions, may reflect different cardiac injuries in patients with COVID-19, which are associated with poor prognosis in these cases.
